# Intermediate-dose cytarabine or standard-dose cytarabine plus single-dose anthracycline as post-remission therapy in older patients with acute myeloid leukemia: impact on health care resource consumption and outcomes

**DOI:** 10.1038/s41408-021-00551-y

**Published:** 2021-11-13

**Authors:** Jean Galtier, Camille Alric, Emilie Bérard, Thibaut Leguay, Suzanne Tavitian, Audrey Bidet, Eric Delabesse, Jean Baptiste Rieu, Jean-Philippe Vial, François Vergez, Nicolas Lechevalier, Isabelle Luquet, Emilie Klein, Anne-Charlotte de Grande, Audrey Sarry, Arnaud Pigneux, Christian Récher, Sarah Bertoli, Pierre-Yves Dumas

**Affiliations:** 1 CHU Bordeaux, Service d’Hématologie Clinique et de Thérapie Cellulaire, F-33000 Bordeaux, France; 2 Service d’Hématologie, Centre Hospitalier Universitaire de Toulouse, Institut Universitaire du Cancer de Toulouse Oncopole, Toulouse, France; 3 Service d’Epidémiologie, Centre Hospitalier Universitaire de Toulouse, Toulouse, France; 4 CERPOP, Université de Toulouse, Inserm, UPS, Toulouse, France; 5 CHU Bordeaux, Laboratoire d’Hématologie Biologique, F-33000 Bordeaux, France; 6 Laboratoire d’Hématologie Biologique, Centre Hospitalier Universitaire de Toulouse, Institut Universitaire du Cancer de Toulouse Oncopole, Toulouse, France; 7 Cancer Research Center of Toulouse, UMR1037 INSERM, ERL5294 CNRS, Toulouse, France; 8 Institut National de la Santé et de la Recherche Médicale, U1035, 33000 Bordeaux, France

**Keywords:** Acute myeloid leukaemia, Acute myeloid leukaemia

Dear Editor,

The treatment of older patients with newly diagnosed acute myeloid leukemia (AML) depends on their fitness. Fit patients receive an induction chemotherapy similar to that of younger patients to achieve complete remission (CR). In patients <60, post-remission treatment is based on repeated courses of intermediate- to high-dose cytarabine with or without allogeneic stem cell transplantation (SCT) according to relapse risk. For patients over 60, there is no consensus about such a strategy, and ELN recommendations suggest intermediate-dose cytarabine (IDAC) for 2–3 cycles in favorable-risk genetics, i.e., 20% of patients. For the remaining 80%, the value of intermediate dose compared to lower-dose cytarabine has not been demonstrated to date, so there is no recommendation in this setting. Nevertheless, IDAC is routinely used, especially in patients selected for allogeneic SCT or as a standard comparator in clinical trials [1]. The IDAC regimen has been adapted to find a compromise between efficacy and toxicity from the results of the Cancer and Leukemia Group B (CALGB) phase 3 trial [2]. Various trials or retrospective studies compared intensive and nonintensive post-remission schedules but none with classical single agent IDAC consolidation [3–7]. In this bi-center retrospective study, we compared the efficacy, safety, and health care resource consumption of two post-remission schedules: IDAC as single agent versus standard-dose cytarabine and single-dose anthracycline (SDAC-IDA) in a large real-world cohort of AML patients.

This study included patients ≥60 years of age with newly diagnosed de novo or secondary AML [8], excluding acute promyelocytic leukemia, in CR or CR with incomplete hematological recovery (CRi) after one course of intensive induction chemotherapy, who received at least one cycle of chemotherapy as post-remission strategy between January 1, 2007, and May 31, 2017. Post-remission strategy consisted in two schedules: one to three cycles of inpatient cytarabine 1.5 g/m^2^ every 12 h for 3 days (9 g/m^2^), referred to as the IDAC arm; versus an outpatient schedule with six courses of idarubicin 8 mg/m²/day IV on day 1 and cytarabine 50 mg/m²/12 h/day subcutaneously on days 1–5 with or without lomustine 40 mg orally on day 1, referred to as the SDAC-IDA arm [9]. Details about Methods are described in Supplementary files.

Of 2905 patients with newly diagnosed AML included in the DATAML registry between 2007 and 2017, a total of 395 AML patients fulfilled the inclusion criteria (Supplementary Fig. 1): 82 (20.8%) in the IDAC arm and 313 (79.2%) in the SDAC-IDA arm. Characteristics of these 395 AML patients are described in Supplementary Table 1. Main significant differences between the IDAC and the SDAC-IDA arms were median age (64.8 y, IQR, 61.8–67.3 versus 68.2 y, IQR, 64.7–72.7, *p* < 0.0001); de novo AML (*n* = 53, 64.6% versus *n* = 256, 81.8%, *p* < 0.0001), and therapy-related AML (*n* = 15, 18.3% versus *n* = 8, 2.6%, *p* < 0.0001). Patients in the SDAC-IDA arm more frequently had an intermediate cytogenetic risk (85.3% versus 51.2%, *p* < 0.0001).

One, two, or three cycles of IDAC were performed in 10 (12.2%), 26 (31.7%), and 46 (56.1%) patients, respectively, in the IDAC arm. Patients received 1–7 cycles of SDAC-IDA in 53 (16.9%), 45 (14.4%), 32 (10.2%), 12 (3.8%), 21 (6.7%), 79 (25.3%), and 71 (22.7%) patients in the SDAC-IDA arm, respectively. The median number of consolidation courses was 3 (IQR 2–3) in the IDAC arm and 5 (IQR 2–6) in the SDAC-IDA arm. Performance status, renal function and weight loss during both consolidation programs are described in Supplementary Table 2. Significantly more patients received an allogeneic SCT in the IDAC arm (*n* = 27, 32.9%) than in SDAC-IDA arm (*n* = 35, 11.2%) (*p* < 0.0001).

Considering the whole population of patients receiving consolidation, the overall incidence rate of infection requiring intravenous antibiotics during the whole post-remission program was 44.1%. Sixty-one (74.4%) patients in the IDAC arm and 113 (36.1%) in the SDAC-IDA arm experienced at least one infection (*p* < 0.0001). Thirty-four (41.5%) patients in the IDAC arm and 49 (15.7%) in the SDAC-IDA arm experienced at least one episode of bacteriemia (*p* < 0.0001). The incidence of microbiologically documented bacteremia by cycle is described in Table 1. Such deep differences have also been observed in incidence of febrile neutropenia without infectious outbreak, overall grade 3–4 infectious events, mean numbers of bacteremia per patient and mean number of infections requiring intravenous antibiotic per patient for whole post-remission program, whereas the median number of consolidation courses was 3 in the IDAC arm and 5 in the SDAC-IDA arm (Table 1). Of note, no neurological toxicities were reported in both arms.

**Table 1:** Impact of post-remission schedule on infections and health care resource consumption.

	IDAC *n* = 82 (20.8%)	SDAC-IDA *n* = 313 (79.2%)	*p* value
Microbiologically documented bacteremia			
Cycle 1—*n* (%)	11 (13.4)	27 (8.7)	0.20
Cycle 2—*n* (%)	20 (27.8)	14 (5.5)	<0.0001
Cycle 3—*n* (%)	7 (15.6)	2 (1.0)	<0.0001
Cycle 4—*n* (%)	–	7 (3.9)	–
Cycle 5—*n* (%)	–	2 (1.2)	–
Cycle 6—*n* (%)	–	1 (0.7)	–
Cycle 7—*n* (%)	–	0 (0.0)	–
Febrile neutropenia without infectious outbreak			
Cycle 1—*n* (%)	32 (39.0)	36 (11.6)	<0.0001
Cycle 2—*n* (%)	31 (43.1)	10 (3.9)	<0.0001
Cycle 3—*n* (%)	14 (30.4)	3 (1.4)	<0.0001
Cycle 4—*n* (%)	–	6 (3.4)	–
Cycle 5—*n* (%)	–	2 (1.2)	–
Cycle 6—*n* (%)	–	1 (0.7)	–
Cycle 7—*n* (%)	–	0 (0.0)	–
Grade 3–4 infections			
Cycle 1—*n* (%)	39 (47.6)	76 (24.5)	<0.0001
Cycle 2—*n* (%)	39 (54.2)	32 (12.5)	<0.0001
Cycle 3—*n* (%)	17 (37.0)	8 (3.8)	<0.0001
Cycle 4—*n* (%)	–	15 (8.4)	–
Cycle 5—*n* (%)	–	3 (1.8)	–
Cycle 6—*n* (%)	–	5 (3.4)	–
Cycle 7—*n* (%)	–	0 (0.0)	–
Cases of bacteremia per patient^a^			
Mean	0.46	0.17	<0.0001
Range	0.0–3.0	0.0–2.0	
Infection requiring intravenous antibiotics per patient^a^			
Mean	1.16	0.44	<0.0001
Range	0.0–3.0	0.0–3.0	
Patients requiring red-cell transfusions—*n*(%)			
Cycle 1	74 (91.4)	181 (60.9)	<0.0001
Cycle 2	63 (76.8)	123 (41.0)	<0.0001
Cycle 3	38 (47.5)	81 (26.9)	<0.0001
Cycle 4	–	66 (21.9)	–
Cycle 5	–	66 (21.9)	–
Cycle 6	–	40 (13.1)	–
Cycle 7	–	20 (6.5)	–
Red-cell transfusions per patient (unit)^a^			
Median	8.0	4.0	<0.0001
Range	0.0–18.0	0.0–24.0	
Patients requiring platelet transfusions—*n*(%)			
Cycle 1	78 (96.3)	182 (61.3)	<0.0001
Cycle 2	67 (81.7)	133 (44.3)	<0.0001
Cycle 3	42 (52.5)	101 (33.8)	0.002
Cycle 4	–	80 (26.7)	–
Cycle 5	–	77 (25.5)	–
Cycle 6	–	60 (19.7)	–
Cycle 7	–	16 (5.2)	–
Platelet transfusions per patient (unit)^a^			
Median	6.0	3.0	<0.0001
Range	0.0–19.0	0.0–23.0	
Length in hospital (days)—Median (IQR)			
Cycle 1	11.0 (10.0–14.0)	4.0 (2.0–8.0)	<0.0001
Cycle 2	12.5 (10.5–16.0)	2.0 (1.0–3.0)	<0.0001
Cycle 3	11.0 (10.0–14.5)	2.0 (1.0–3.0)	<0.0001
Cycle 4	–	2.0 (1.0–3.0)	–
Cycle 5	–	2.0 (1.0–2.0)	–
Cycle 6	–	2.0 (1.0–2.0)	–
Cycle 7	–	1.0 (1.0–2.0)	–
Length in hospital per patient (days)^a^			
Median	32.5	12.0	<0.0001
Range	9.0–64.0	0.0–68.0	

*IDAC* intermediate-dose cytarabine, *SDAC-IDA* standard-dose cytarabine and single-dose idarubicin, *IQR* interquartile range.

^a^For whole post-remission program.

Considering the whole post-remission program, the median red blood cell transfusion per patient was 8.0 units (range, 0.0–18.0) in the IDAC arm and 4.0 (range, 0.0–24.0) in the SDAC-IDA arm (*p* < 0.0001). Considering the whole post-remission program, the median platelet transfusion per patient was 6.0 units (range, 0.0–19.0) in the IDAC arm and 3.0 (range, 0.0–23.0) in the SDAC-IDA arm (*p* < 0.0001). These red blood cell and platelet transfusions consumptions are described by cycle in Table 1. Finally, despite hospitalization for transfusion, infectious complications, and a post-remission program twice as long, the length in hospital for the whole post-remission schedule per patient was a median of 12.0 days (range, 0.0–68.0) in the SDAC-IDA arm versus 32.5 days (range, 9.0–64.0) in the IDAC arm (*p* < 0.0001). Length in hospital is also detailed in Table 1. Per cycle, patients in the IDAC arm were hospitalized for a median of 12.5 days (IQR, 10.8–14.2) versus a median of 3.0 days (IQR, 2.0–4.8) in the SDAC-IDA arm (*p* < 0.0001).

The median follow‐up periods were 64.1 months (IQR, 53.9–74.3) in the IDAC arm and 73.8 months (IQR, 57.9–103.8) in the SDAC-IDA arm. The median overall survival (OS) from diagnosis was 39.7 months [IQR, 14.2-not reached (NR)] in the IDAC arm and 30.3 months (IQR, 14.3–84.1) in the SDAC-IDA arm (Fig. 1A) (*p* = 0.162). The median relapse-free survival (RFS) was 20.9 months (IQR, 6.0-NR) in the IDAC arm and 17.5 months (IQR, 7.9–75.0) in the SDAC-IDA arm (Fig. 1B) (*p* = 0.187). Five-year cumulative incidence of relapse (CIR) was 47% (95% CI 53.0–63.0) in the IDAC arm and 65% (95% CI 56.0–66.0) in the SDAC-IDA arm (Fig. 1C) (*p* = 0.050). Five-year nonrelapse mortality (NRM) was 15% (95% CI 7.0–13.0) in the IDAC arm and 9% (95% CI 8.0–14.0) in the SDAC-IDA arm (Fig. 1D) (*p* = 0.115). After adjusting for center, factors significantly and independently associated with OS, RFS, CIR, and NRM are described in Supplementary Table 3. Overall, after adjustment, post-remission schedule by IDAC or SDAC-IDA was not significantly and independently associated with all outcomes tested, (including CIR), whereas allogeneic SCT was associated with better outcome (HR = 0.49 for OS and 0.46 for RFS, *p* < 0.001 for both). Interactions between all potential confounding factors and IDAC versus SDAC-IDA were tested in survival and relapse models. None were significant, indicating that the effect of IDAC versus SDAC-IDA regimen was not significantly different in OS, RFS, CIR, or NRM according to all confounding factors analyzed, especially according to ELN 2010 prognosis, de novo or secondary AML, allogeneic SCT, and age (Supplementary Figs. 2, 3, 4 and 5).

**Fig. 1:**
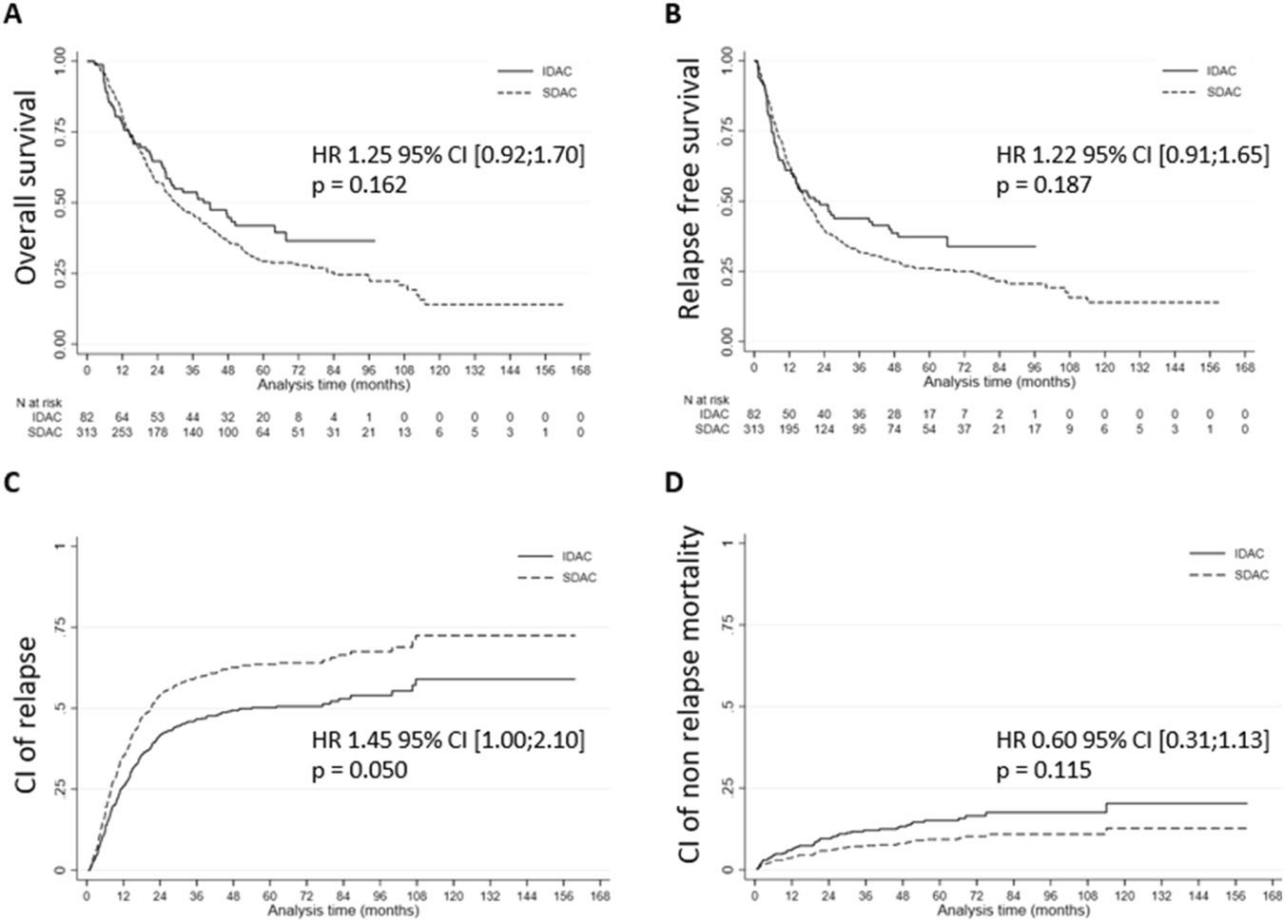
Outcomes among 395 patients over 60 with acute myeloid leukemia who achieved complete remission/complete remission with incomplete hematological recovery after an intensive first induction course. **A** Overall survival by treatment arm; **B** Relapse-free survival by treatment arm; **C** Cumulative incidence of relapse by treatment arm; **D** Cumulative incidence of nonrelapse mortality by treatment arm. HR hazard ratio, CI confidence interval.

This study demonstrated considerable differences in terms of infectious events, red blood cell transfusions, platelet transfusions, and length of hospitalization. Patients receiving the SDAC-IDA regimen spent on average 20 days less in hospital over the whole period of post-remission treatment. The IDAC regimen was also associated with a higher rate of bacteremia and febrile neutropenia. We also observed that both IDAC or SDAC-IDA regimens were associated with similar OS, RFS, and CIR whereas allogeneic HSCT was significantly and independently associated with these outcomes. Obviously, the main drawback of this study is the lack of randomization and the absence of pre-established criteria for selecting treatments once complete remission was achieved. In our routine practice and in our cooperative group (FILO), we generally use the SDAC-IDA schedule. However, IDAC was occasionally performed to patients included in clinical trials or those with a favorable-risk profile in order to increase dose intensity in chemosensitive disease, and to adverse-risk patients in order to reduce residual disease to a minimum before transplantation. To address these discrepancies between the IDAC and SDAC-IDA groups, we did multivariate analyses and interaction tests but did not find any impact of treatment intensity.

In conclusion, the results of this study do not demonstrate that SDAC-IDA does as well as IDAC in this situation due to its retrospective design, we can nevertheless conclude that the use of health care resources, including length of hospitalization, is considerably reduced with SDAC-IDA. This has a major impact on treatment costs and quality of life. In addition, blood products and hospital beds can be saved with this treatment. Except CBF AML, we will therefore continue to provide SDAC-IDA to our patients until new, more effective and hopefully less toxic strategies become available [10].

## Supplementary information

Supplementary methods

Supplementary tables

Supplementary figures
