# Supplementary material for: Intermediate-dose cytarabine or standard-dose cytarabine plus single-dose anthracycline as post-remission therapy in older patients with acute myeloid leukemia: impact on health care resource consumption and outcomes
Source: Blood Cancer J. 2021 Nov 13;11(11):180. doi: 10.1038/s41408-021-00551-y (PMC8590686; doi:10.1038/s41408-021-00551-y)
Supplement: Supplementary file 1 — Supplementary methods [file 41408_2021_551_MOESM1_ESM.docx]

**Intermediate-dose cytarabine or standard-dose cytarabine plus single-dose anthracycline as post-remission therapy in older patients with acute myeloid leukemia: impact** **on health care resource consumption and outcomes**

**Supplementary Methods**

**Patients and treatments**

The prophylactic use of granulocyte colony-stimulating factor (G-CSF) was systematic, either the pegylated formulation of G-CSF, pegfilgrastim (6 mg) or the standard daily dose of G-CSF (5 μg/kg/day). In our practice, patients are given IDAC treatment on an inpatient basis and are then discharged after chemotherapy, then re-admitted systematically for management of myelosuppression on day 10-12 according to their blood cell count. Conversely, patients in the SDAC-IDA arm receive chemotherapy in an outpatient setting and are hospitalized only if they need transfusion or in the event of febrile neutropenia. None of them received antibiotic prophylaxis. Primary refractory AML was defined as a failure to achieve CR1/CRi1 after one induction chemotherapy, and such cases were not included in the current study. The study was conducted in accordance with the Declaration of Helsinki, allowing the collection of clinical data in the anonymized French Toulouse-Bordeaux DATAML registry.

Cytogenetic risk classification was defined according to the UK MRC classification ^1^ and comorbidities were defined according to Charlson Comorbidity Index ^2^. Intensive induction chemotherapy was based on idarubicin at a daily dose of 8 mg/m^2^ for 5 days or daunorubicin at a daily dose of 60 mg/m^2^ for 3 days, together with continuous intravenous infusion of cytarabine at a daily dose of 100–200 mg/m^2^ for 7 days, with or without lomustine 200 mg/m² at day 1 ^3^. Bone marrow assessment was performed in patients treated with intensive chemotherapy after hematological recovery or between days 35 and 45 if recovery was delayed. Allogeneic SCT was performed until 70 years old after one or two consolidation cycles in most patients. Response to treatment, relapse, RFS, cumulative incidence of relapse (CIR), non-relapse mortality (NRM), and OS were defined according to the ELN criteria ^4^. Bacteremia was defined as the presence of bacteria in the blood, grade 3-4 infections were defined according to CTCAE v4.0, neutropenia was defined as an absolute neutrophil count below 0.5 × 10^9^/L, and length in hospital stay included days of hospitalization for chemotherapy, infection, or day hospital for transfusion. Each day started was counted.

**Statistical analysis**

The patients’ characteristics are described using numbers and frequencies for qualitative data, and median, interquartile range (IQR), and range (minimum − maximum) for quantitative data. Categorical variables were compared between IDAC and SDAC-IDA arms using the χ^2^ test (or Fisher’s exact test when necessary). Student’s *t* test was used to compare the distributions of continuous data (Mann–Whitney’s test was used when the distribution departed significantly from normality or when homoscedasticity was rejected). For RFS and OS, differences in survival functions between the IDAC and SDAC-IDA arms were described using median with IQR and were tested using the log-rank test. For relapse (CIR) and NRM, cumulative incidence functions were drawn (as NRM or relapse was used as a competing event) and compared using Gray’s test. Adjusted hazard ratios (aHR) and 95% confidence intervals (95%CI) were assessed using a standard Cox model for RFS and OS, and a proportional sub-distribution hazard model (an extension of the Cox model) for competing risks for CIR and NRM ^5^. Multivariate analyses included IDAC *vs* SDAC-IDA together with potential confounding factors [center, ELN 2010 prognosis, AML status (*de novo* or secondary AML), age, performance status, WBC at diagnosis, ferritinemia and albuminemia at diagnosis, delay between first induction course and consolidation cycle 1 and allogeneic SCT in CR1/CRi1]. Stepwise regression analysis was then used to assess variables that were significantly and independently associated with the endpoints (*p* < 0.05). The proportional hazard assumption was tested for each covariate of the Cox model using log-log plot curves and was always supported. When the linear hypothesis was not supported, continuous potential confounding factors were transformed into ordered data. Interactions between all potential confounding factors and IDAC *vs* SDAC-IDA were tested. None were significant, indicating that the effect of treatment (IDAC *vs* SDAC-IDA) was not significantly different according to all confounding factors analyzed, especially according to age, ELN 2010 prognosis, AML status (*de novo* or secondary AML) and allogeneic SCT. Allogeneic SCT in CR1/CRi1 was evaluated as a time-dependent qualitative covariate. All reported *p*-values were two-sided, and the significance threshold was *p*<0.05. Statistical analyses were performed using STATA® version 14.2 (STATA Corp., College Station, TX).

**References**

1 Grimwade D, Walker H, Oliver F, Wheatley K, Harrison C, Harrison G et al. The importance of diagnostic cytogenetics on outcome in AML: analysis of 1,612 patients entered into the MRC AML 10 trial. The Medical Research Council Adult and Children’s Leukaemia Working Parties. Blood 1998; 92: 2322–2333.

2 Charlson ME, Pompei P, Ales KL, MacKenzie CR. A new method of classifying prognostic comorbidity in longitudinal studies: development and validation. J Chronic Dis 1987; 40: 373–383.

3 Pigneux A, Béné MC, Salmi L-R, Dumas P-Y, Delaunay J, Bonmati C et al. Improved Survival by Adding Lomustine to Conventional Chemotherapy for Elderly Patients With AML Without Unfavorable Cytogenetics: Results of the LAM-SA 2007 FILO Trial. J Clin Oncol Off J Am Soc Clin Oncol 2018; 36: 3203–3210.

4 Döhner H, Estey EH, Amadori S, Appelbaum FR, Büchner T, Burnett AK et al. Diagnosis and management of acute myeloid leukemia in adults: recommendations from an international expert panel, on behalf of the European LeukemiaNet. Blood 2010; 115: 453–474.

5 Fine J, Gray R. A Proportional Hazards Model for the Subdistribution of a Competing Risk. J Am Stat Assoc; 1999;94(446):496–509.
