# Supplementary material for: Intermediate-dose cytarabine or standard-dose cytarabine plus single-dose anthracycline as post-remission therapy in older patients with acute myeloid leukemia: impact on health care resource consumption and outcomes
Source: Blood Cancer J. 2021 Nov 13;11(11):180. doi: 10.1038/s41408-021-00551-y (PMC8590686; doi:10.1038/s41408-021-00551-y)
Supplement: Supplementary file 2 — Supplementary tables [file 41408_2021_551_MOESM2_ESM.docx]

**Supplementary Table 1. Characteristics at diagnosis of 395 AML patients who achieved CR1/CRi1 after intensive first induction course**

|  | **IDAC**  **n = 82 (20.8%)** | **SDAC-IDA**  **n = 313 (79.2%)** | ***p*-value** | **Total**  **n = 395 (100%)** |
| --- | --- | --- | --- | --- |
| **Age at diagnosis (years)**  Median (IQR)  Range | 64.8 (61.8-67.3)  60.3-73.6 | 68.2 (64.7-72.7)  60.1-80.9 | <0.0001 | 67.3 (64.0-71.5)  60.1-80.9 |
| **Sex: n (%)**  Man  Woman | 46 (56.1)  36 (43.9) | 195 (62.3)  118 (37.7) | 0.31 | 241 (61.0)  154 (39.0) |
| **ECOG at diagnosis: n (%)**  0–1  ≥ 2 | 69 (85.2)  12 (14.8) | 246 (81.7)  55 (18.3) | 0.47 | 315 (82.5)  67 (17.5) |
| **Comorbidity index: n (%)**  0  1  ≥ 2 | 52 (64.2)  14 (17.3)  15 (18.5) | 196 (64.1)  63 (20.6)  47 (15.4) | 0.77 | 248 (64.1)  77 (19.9)  62 (16.0) |
| **WBC at diagnosis (×10^9^/L)**  Median (IQR)  Range | 4.2 (2.1-12.1)  0.7-239.9 | 5.4 (2.1-33.0)  0.4-359.4 | 0.21 | 4.9 (2.1-28.2)  0.4-359.4 |
| **AML status: n (%)**  *De novo*  Secondary AML^a^ | 53 (64.6)  29 (35.4) | 256 (81.8)  57 (18.2) | <0.0001 | 309 (78.2)  86 (21.8) |
| **Cytogenetic risk: n (%)**  Favorable  Intermediate  Adverse | 14 (17.1)  42 (51.2)  26 (31.7) | 10 (3.2)  267 (85.3)  36 (11.5) | <0.0001 | 24 (6.1)  309 (78.2)  62 (15.7) |
| **ELN 2010 prognosis: n (%)**  Favorable  Intermediate-I/II  Adverse | 22 (28.9)  28 (36.9)  26 (34.2) | 61 (22.3)  177 (64.6)  36 (13.1) | <0.0001 | 83 (23.7)  205 (58.6)  62 (17.7) |
| ***FLT3-*ITD: n (%)**  Mutation  No mutation | 5 (10.6)  42 (89.4) | 52 (21.6)  189 (78.4) | 0.09 | 57 (19.8)  231 (80.2) |
| ***NPM1*: n (%)**  Mutation  No mutation | 15 (31.9)  32 (68.1) | 99 (41.4)  140 (58.6) | 0.22 | 114 (39.9)  172 (60.1) |
| ***IDH1 R132*: n (%)**  Mutation  No mutation | 3 (15.0)  17 (85.0) | 9 (11.0)  73 (89.0) | 0.70 | 12 (11.8)  90 (88.2) |
| ***IDH2 R140*: n (%)**  Mutation  No mutation | 1 (5.0)  19 (95.0) | 15 (17.6)  70 (82.4) | 0.30 | 16 (15.2)  89 (84.8) |
| ***IDH2 R172*: n (%)**  Mutation  No mutation | 4 (20.0)  16 (80.0) | 2 (2.4)  83 (97.6) | 0.01 | 6 (5.7)  99 (94.3) |
| **Induction course: n (%)**  Daunorubicin-based  Idarubicin-based  w/ CCNU | 10 (12.2)  71 (86.6)  62 (75.6) | 3 (1.0)  306 (97.8)  221 (70.6) | <0.0001 | 13 (3.3)  377 (95.4)  283 (71.6) |

CR, complete remission; CRi, complete remission with incomplete hematologic recovery; IDAC, intermediate-dose cytarabine; SDAC-IDA, standard-dose cytarabine and single dose idarubicin; AML, acute myeloid leukemia; ECOG, performance status; ELN, European Leukemia Net; IQR, interquartile range; ITD, internal tandem duplication; WBC, white blood cells; w/ CCNU, with lomustine.

^a^ Non-*de novo* AML (MDS, MPN, therapy-related)

**Supplementary Table 2. Baseline renal function, weight, and performance status at each consolidation cycle**

|  | **IDAC**  **n = 82 (20.8%)** | **SDAC-IDA**  **n = 313 (79.2%)** | ***p*-value** |
| --- | --- | --- | --- |
| **Creatinine at day 1 of each cycle (µM/L)**  Cycle 1 - Median (IQR)  Cycle 2 - Median (IQR)  Cycle 3 - Median (IQR)  Cycle 4 - Median (IQR)  Cycle 5 - Median (IQR)  Cycle 6 - Median (IQR)  Cycle 7 - Median (IQR) | 69.0 (61.0-82.0)  71.0 (60.0-83.5)  70.0 (58.0-82.0)  -  -  -  - | 75.0 (65.0-90.0)  76.0 (63.0-90.0)  78.0 (66.0-92.5)  80.0 (65.0-97.0)  78.0 (65.0-95.0)  73.5 (65.0-90.0)  79.0 (64.0-97.0) | 0.024  0.047  0.006  -  -  -  - |
| **Weight at day 1 of each cycle (kg)**  Cycle 1 - Median (IQR)  Cycle 2 - Median (IQR)  Cycle 3 - Median (IQR)  Cycle 4 - Median (IQR)  Cycle 5 - Median (IQR)  Cycle 6 - Median (IQR)  Cycle 7 - Median (IQR) | 71.0 (63.0-80.0)  74.0 (67.0-82.0)  69.5 (59.0-80.0)  -  -  -  - | 70.0 (60.0-79.5)  70.0 (60.0-80.0)  70.0 (60.0-80.0)  69.5 (59.0-80.0)  72.0 (59.0-80.0)  70.0 (58.3-80.0)  67.5 (58.5-77.0) | 0.75  0.59  0.57  -  -  -  - |
| **ECOG 0 at day 1 of each cycle**  Cycle 1 - n (%)  Cycle 2 - n (%)  Cycle 3 - n (%)  Cycle 4 - n (%)  Cycle 5 - n (%)  Cycle 6 - n (%)  Cycle 7 - n (%) | 26 (41.3)  24 (40.0)  21 (53.8)  -  -  -  - | 48 (18.0)  58 (26.9)  59 (32.6)  54 (35.3)  41 (28.7)  47 (36.2)  21 (33.3) | <0.001  0.104  0.04  -  -  -  - |

IDAC, intermediate-dose cytarabine; SDAC, standard-dose cytarabine; IQR, interquartile range; ECOG, performance status

**Supplementary Table 3. Cox model* for factors independently associated with overall survival, relapse-free survival, cumulative incidence of relapse and non-relapse mortality**

|  | **n** | **Events** | **aHR** | **^95%^CI** | ***p*-value** |
| --- | --- | --- | --- | --- | --- |
| **Overall survival** | | | | | |
| **Consolidation regimen**  IDAC  SDAC-IDA | 82  313 | 49  229 | 1  1.18 | -  0.83-1.68 | -  0.342 |
| **ELN 2010 prognosis**  Favorable  Intermediate  Adverse | 83  205  62 | 50  150  45 | 1  1.41  2.05 | -  1.01-1.97  1.34-3.15 | -  0.040  0.001 |
| **Comorbidity index**  0-1  ≥ 2 | 325  62 | 222  49 | 1  1.39 | -  1.01-1.91 | -  0.043 |
| **Ferritinemia at diagnosis (µg/L)**  < 384 µg/L  ≥ 384 µg/L | 76  228 | 48  162 | 1  1.50 | -  1.08-2.10 | -  0.015 |
| **Delay between induction and consolidation n°1 (days)**  < 59 days  ≥ 59 days | 305  90 | 209  69 | 1  1.60 | -  1.18-2.17 | -  0.002 |
| **Allogeneic SCT**  No  Yes | 333  62 | 248  30 | 1  0.48 | -  0.32-0.74 | -  0.001 |
| **Relapse-free survival** | | | | | |
| **Consolidation regimen**  IDAC  SDAC-IDA | 82  313 | 52  239 | 1  1.19 | -  0.85-1.66 | -  0.312 |
| **ELN 2010 prognosis**  Favorable  Intermediate  Adverse | 83  205  62 | 57  155  45 | 1  1.21  1.61 | -  0.88-1.65  1.06-2.44 | -  0.236  0.024 |
| **Delay between induction and consolidation n°1 (days)**  < 59 days  ≥ 59 days | 305  90 | 219  72 | 1  1.54 | -  1.15-2.07 | -  0.004 |
| **Allogeneic SCT**  No  Yes | 333  62 | 261  30 | 1  0.46 | -  0.30-0.70 | -  <0.001 |
| **Cumulative incidence of relapse** | | | | | |
| **Consolidation regimen**  IDAC  SDAC-IDA | 82  313 | 39  208 | 1  1.25 | -  0.84-1.85 | -  0.264 |
| **ELN 2010 prognosis**  Favorable  Intermediate  Adverse | 83  205  62 | 48  133  36 | 1  1.28  1.76 | -  0.91-1.80  1.06-2.91 | -  0.153  0.028 |
| **Allogeneic SCT**  No  Yes | 333  62 | 229  18 | 1  0.30 | -  0.18-0.51 | -  <0.001 |
| **Non-relapse mortality** | | | | | |
| **Consolidation regimen**  IDAC  SDAC-IDA | 82  313 | 13  31 | 1  0.94 | -  0.43-2.18 | -  0.940 |
| **Allogeneic SCT**  No  Yes | 333  62 | 32  12 | 1  2.78 | -  1.27-6.06 | -  0.010 |

*Adjusted for center

IDAC, intermediate-dose cytarabine; SDAC-IDA, standard-dose cytarabine and single dose idarubicin; aHR, adjusted hazard ratio; CI, confidence interval; ELN, European Leukemia Net; SCT, stem cell transplantation.

Interactions between treatment (IDAC *versus* SDAC-IDA) and all potential confounding factors analyzed *[center, ELN 2010 prognosis, AML status (de novo or secondary AML), age, performance status, WBC at diagnosis, ferritinemia and albuminemia at diagnosis, delay between first induction course and consolidation cycle 1 and allogeneic SCT in CR1/CRi1]* were not significant, indicating that effect of treatment (IDAC *versus* SDAC-IDA) is not significantly different according to all confounding factors analyzed, in particular according to age, ELN 2010 prognosis, AML status (de novo or secondary AML) or allogeneic SCT [see supplementary Figure 2 to 5].
