# Supplementary material for: Intermediate-dose cytarabine or standard-dose cytarabine plus single-dose anthracycline as post-remission therapy in older patients with acute myeloid leukemia: impact on health care resource consumption and outcomes
Source: Blood Cancer J. 2021 Nov 13;11(11):180. doi: 10.1038/s41408-021-00551-y (PMC8590686; doi:10.1038/s41408-021-00551-y)
Supplement: Supplementary file 3 — Supplementary figures [file 41408_2021_551_MOESM3_ESM.pptx]

## Slide 1
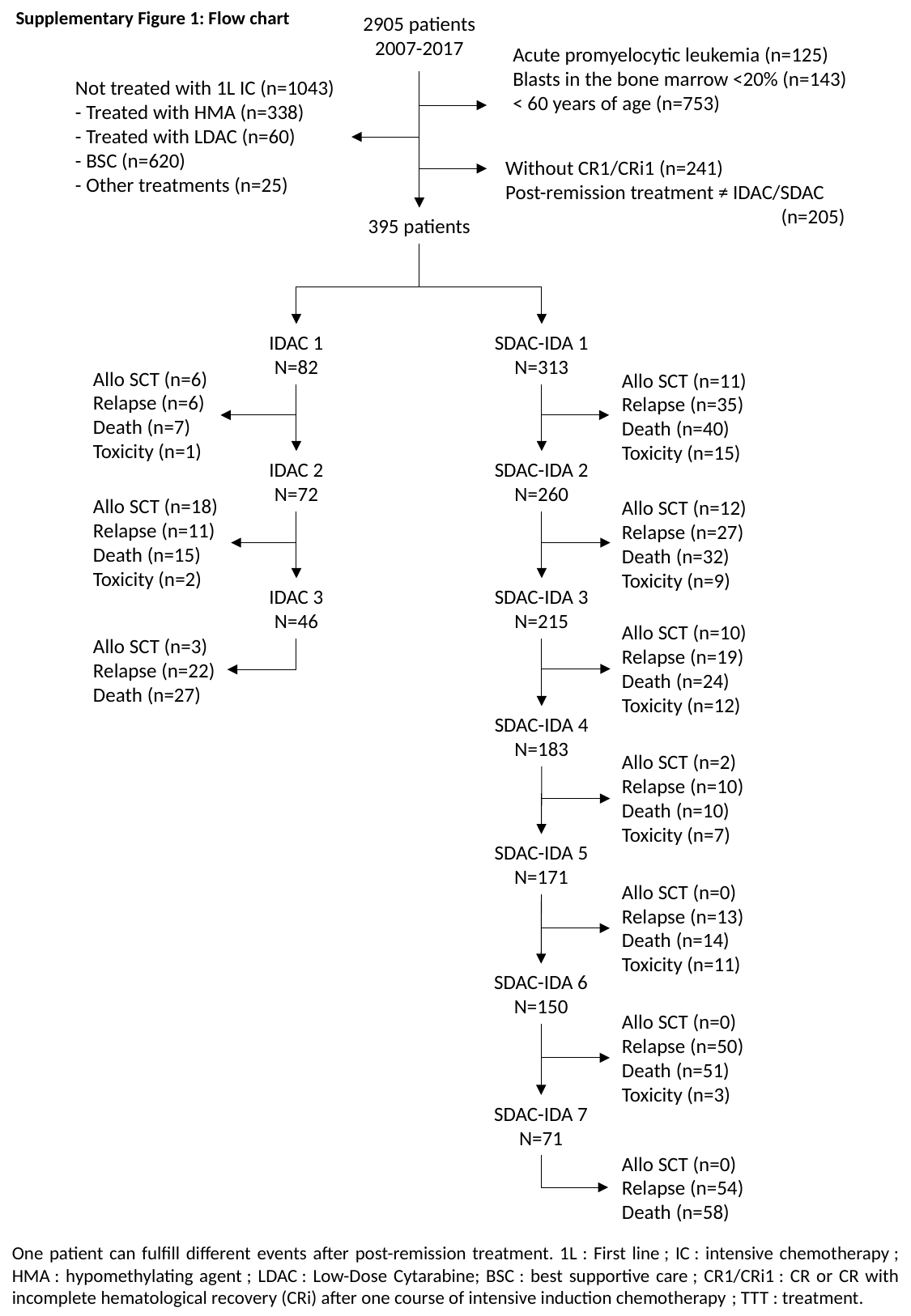

Supplementary Figure 1: Flow chart
2905 patients
2007-2017
Acute promyelocytic leukemia (n=125)
Blasts in the bone marrow <20% (n=143)
< 60 years of age (n=753)
Not treated with 1L IC (n=1043)
- Treated with HMA (n=338)
- Treated with LDAC (n=60)
- BSC (n=620)
- Other treatments (n=25)
Without CR1/CRi1 (n=241)
Post-remission treatment ≠ IDAC/SDAC
		 (n=205)
395 patients
IDAC 1
N=82
SDAC-IDA 1
N=313
Allo SCT (n=6)
Relapse (n=6)
Death (n=7)
Toxicity (n=1)
Allo SCT (n=11)
Relapse (n=35)
Death (n=40)
Toxicity (n=15)
IDAC 2
N=72
SDAC-IDA 2
N=260
Allo SCT (n=18)
Relapse (n=11)
Death (n=15)
Toxicity (n=2)
Allo SCT (n=12)
Relapse (n=27)
Death (n=32)
Toxicity (n=9)
IDAC 3
N=46
SDAC-IDA 3
N=215
Allo SCT (n=10)
Relapse (n=19)
Death (n=24)
Toxicity (n=12)
Allo SCT (n=3)
Relapse (n=22)
Death (n=27)
SDAC-IDA 4
N=183
Allo SCT (n=2)
Relapse (n=10)
Death (n=10)
Toxicity (n=7)
SDAC-IDA 5
N=171
Allo SCT (n=0)
Relapse (n=13)
Death (n=14)
Toxicity (n=11)
SDAC-IDA 6
N=150
Allo SCT (n=0)
Relapse (n=50)
Death (n=51)
Toxicity (n=3)
SDAC-IDA 7
N=71
Allo SCT (n=0)
Relapse (n=54)
Death (n=58)
One patient can fulfill different events after post-remission treatment. 1L : First line ; IC : intensive chemotherapy ; HMA : hypomethylating agent ; LDAC : Low-Dose Cytarabine; BSC : best supportive care ; CR1/CRi1 : CR or CR with incomplete hematological recovery (CRi) after one course of intensive induction chemotherapy ; TTT : treatment.

## Slide 2
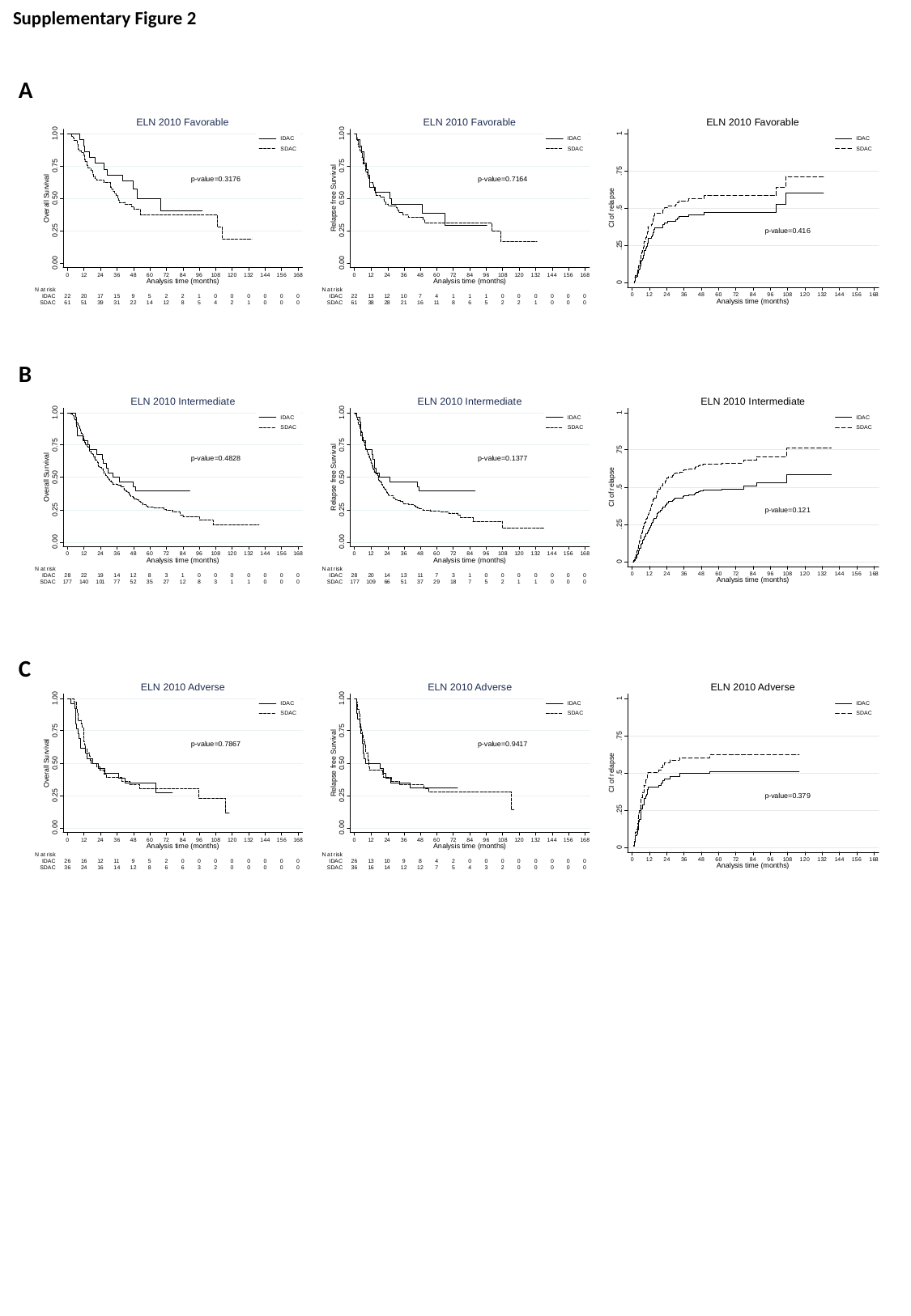

Supplementary Figure 2
A
B
C

## Slide 3
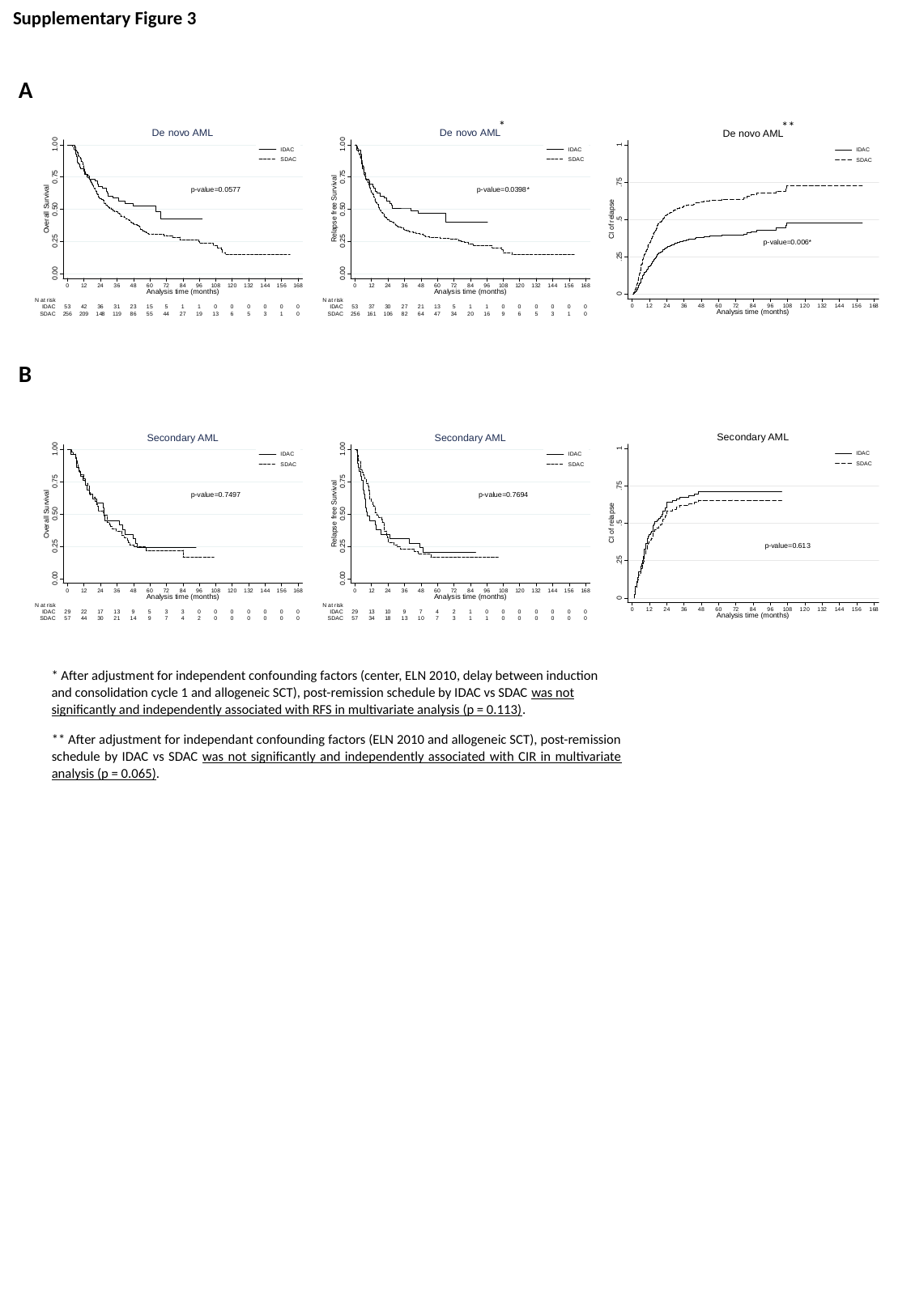

Supplementary Figure 3
A
*
**
B
* After adjustment for independent confounding factors (center, ELN 2010, delay between induction and consolidation cycle 1 and allogeneic SCT), post-remission schedule by IDAC vs SDAC was not significantly and independently associated with RFS in multivariate analysis (p = 0.113).
** After adjustment for independant confounding factors (ELN 2010 and allogeneic SCT), post-remission schedule by IDAC vs SDAC was not significantly and independently associated with CIR in multivariate analysis (p = 0.065).

## Slide 4
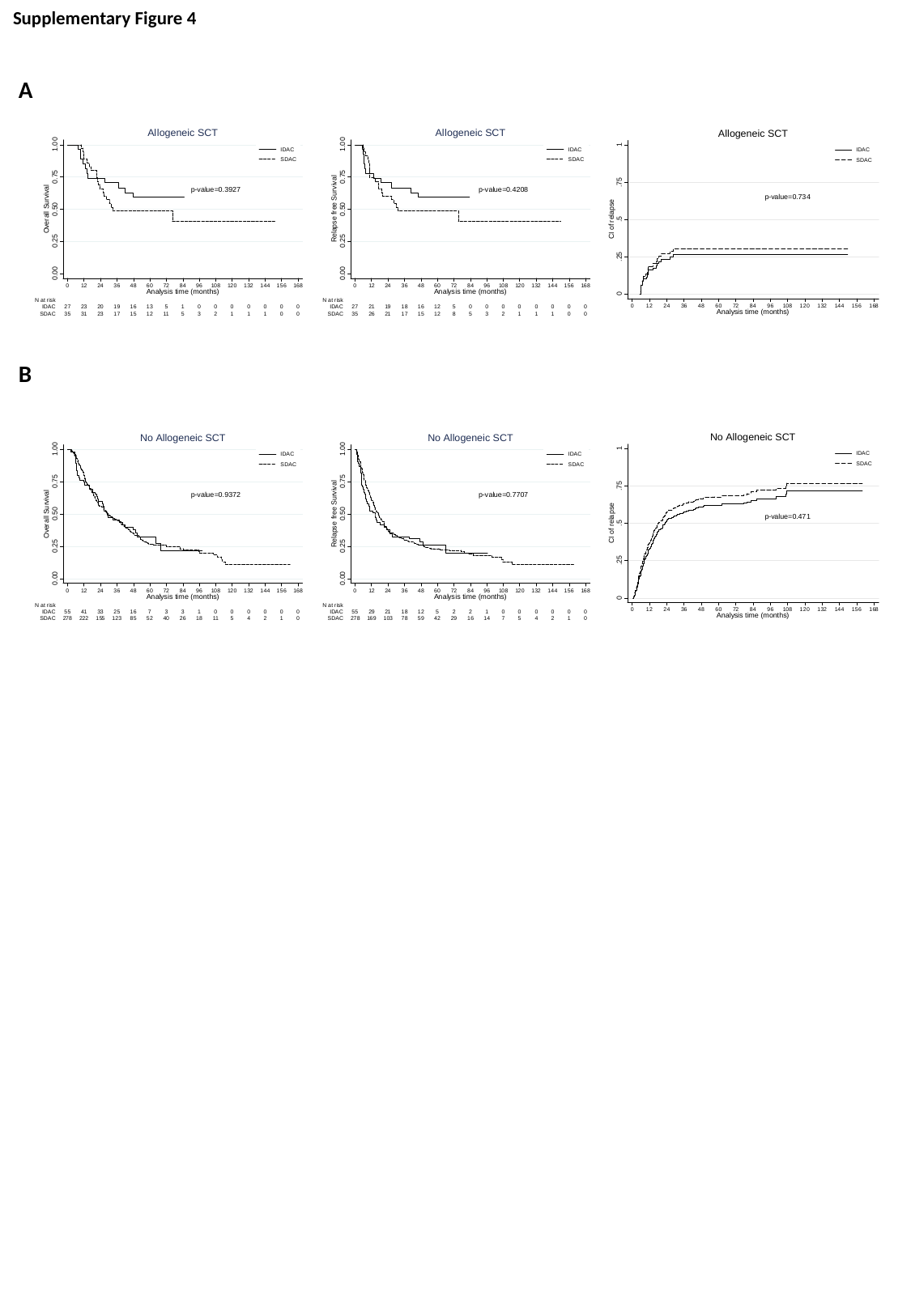

Supplementary Figure 4
A
B

## Slide 5
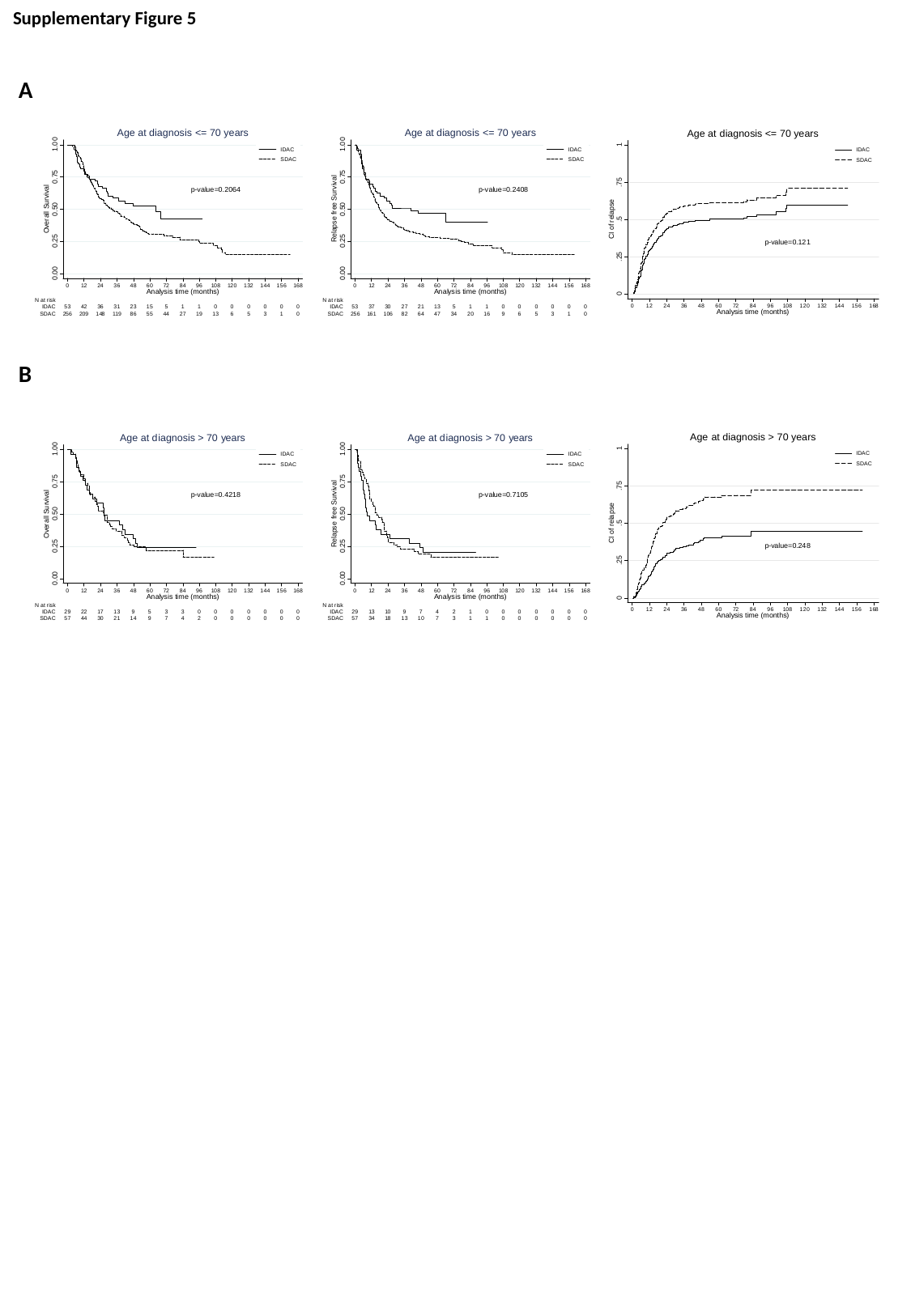

Supplementary Figure 5
A
B
